# Decision-Making concerning Involuntary Oral Care for Older Individuals with Dementia

**DOI:** 10.3390/ijerph192416655

**Published:** 2022-12-11

**Authors:** Maud Jonker, Coos Engelsma, David J. Manton, Anita Visser

**Affiliations:** 1 Department of Gerodontology, Center for Dentistry and Oral Hygiene, University Medical Center Groningen, University of Groningen, 9713 AV Groningen, The Netherlands; 2 Medical Ethics and Decision Making, Department of Ethics, Center for Dentistry and Oral Hygiene, University Medical Center Groningen, University of Groningen, 9713 AV Groningen, The Netherlands; 3 Department of Cariology, Center for Dentistry and Oral Hygiene, University Medical Center Groningen, University of Groningen, 9713 AV Groningen, The Netherlands; 4 Department of Paediatric Dentistry, Academic Centre for Dentistry Amsterdam (ACTA), Vrije Universiteit Amsterdam and University of Amsterdam, 1081 LA Amsterdam, The Netherlands; 5 Department of Gerodontology, Faculty for Dentistry, Radboud University Medical Center, Radboud University Nijmegen, 6525 EX Nijmegen, The Netherlands

**Keywords:** involuntary care, care-resistant behavior, dementia, oral health care, elderly, nursing homes

## Abstract

Many older individuals with dementia (OIWD) show care-resistant behavior for oral care. Providing care despite resistance is considered to be involuntary care. Although involuntary care should be minimized, in some OIWD it may be necessary to avoid health risks. This study aims to assess the attitudes of healthcare providers with regard to involuntary oral care provision for OIWD who show care-resistant behavior. An online questionnaire consisting of general questions and case specific questions was administered via social media and networking to individuals involved in the oral care of OIWD. A total of 309 participants were included in this study. The outcomes of the questionnaires were categorized per case. In all cases, a discrepancy was seen between the assessment of oral health problems as potentially harmful (range: 73.1–93.5%) and the willingness to provide involuntary care (range: 31.1–63.4%). Hence, many healthcare providers are aware of the subsequent potential health risks related to not providing care, but are still reluctant to provide involuntary oral care. Therefore, many OIWD who show care-resistant behavior potentially do not receive the necessary oral care they require.

## 1. Introduction

Because of societal ageing, not only will the number of older people worldwide increase significantly over the next three decades, they will also reach older ages, increasing the risk of developing dementia [1,2]. Almost 40% of individuals aged ninety and older suffer from dementia [3]. Therefore, it is predicted that the number of people with dementia will grow rapidly. The World Alzheimer Report predicts that the number of people with dementia worldwide will triple from 50 million in 2018 to more than 152 million in 2050 [4].

In most individuals diagnosed with dementia, cognitive functions will decline slowly [5], often reducing the ability for self-care and activities of daily living (ADLs), such as bathing, clothing and oral hygiene [6]. Therefore, older individuals with dementia (OIWD) are usually dependent on others for their ADLs. However, many OIWD have impaired ability to understand the intentions of others to provide help and often show care-resistant behavior during ADL care [7]. 

One field in which care-resistant behavior constitutes a significant challenge is dentistry. Most healthcare providers who work with OIWD and assist with their ADLs encounter care-resistant behavior during oral care provision. One study reports that 97% of 86 Swedish nursing home professionals experienced care-resistant behavior during oral care provision [8] and, similarly, another study reports that 97% of 494 nurses in 11 Norwegian nursing homes experienced such resistance during oral care provision [9].

If care is provided in the presence of care-resistant behavior, this is considered to be involuntary care. Involuntary care should be avoided as much as possible, since it generally has a negative psychological impact on patients, often resulting in anger, humiliation, fear, distress and depression [10]. In addition, involuntary care, at least in many situations, is illegal in several countries [11,12,13].

Possible strategies and interventions to avoid or reduce care-resistant behavior in OIWD during (oral) care provision have been studied, and include respectful communication (no use of ‘elderspeak’ or ’baby talk’), the use of distraction (such as music or singing) and a quiet environment to provide care [14,15,16]. These strategies putatively reduce care-resistant behavior, but could not eliminate it for all OIWD [15,16]. 

When all strategies and interventions for voluntary care have been attempted and patients remain resistant, this does not always mean that healthcare providers should cease care provision, as potential detrimental consequences exist. For example, not providing daily oral care can lead to poor oral health and, indirectly, poor general health, which can be associated with conditions such as endocarditis, pneumonia, cardiovascular disease, etc. [17,18,19]. Additionally, poor oral health can lead to pain and discomfort, which is detrimental to an individual’s wellbeing [18]. 

In light of the possible negative consequences of involuntary care as well as the need for involuntary care in specific situations, several countries have developed regulatory laws [11,12,13]. For example, in the Netherlands, a law was promulgated in January 2020, regulating that involuntary care is only allowed when not providing care can lead to serious harm for the patient or other individuals. Providing involuntary care to patients who are mentally incapacitated in the Netherlands is illegal unless specific actions have been undertaken, and all parties involved acknowledge (potential) harm and agree to provide involuntary care as the only way to prevent harm. According to this law, the provision of involuntary care must satisfy three criteria: (1) proportionality: the involuntary care is in reasonable proportion to its purpose, (2) subsidiarity: the least drastic care is provided to achieve the purpose and (3) effectiveness: the involuntary care achieves the intended purpose and does not last longer than strictly necessary [11].

Reaching well-informed decisions about OIWD who need oral care but show care-resistant behavior requires knowledge of the subject. Interestingly, several authors claim that most healthcare providers who provide oral care (such as brushing teeth) as part of ADL care, do not have sufficient knowledge about oral health and how to provide proper oral care [20,21]. Due to limited knowledge of oral health problems, healthcare providers may not consider them potentially harmful, and therefore are not likely to provide involuntary oral care, as they are unaware of the potentially harmful consequences of this inaction. Other potential reasons for not providing involuntary oral care are that it can be unpleasant for healthcare providers as well as for OIWD [8], that it can cause moral and ethical distress in healthcare providers [10], and that it can be difficult to provide because of aggressive patient behavior (verbal or physical) [22].

It is currently unknown how often and in which cases individuals who are involved in the oral healthcare of OIWD provide oral care when a patient shows care-resistant behavior. This is unfortunate, since there is a high prevalence of oral health problems in OIWD, especially those living in nursing homes [23,24] and, importantly, as the provision of oral care can help prevent severe oral health problems. 

Therefore, the aim of this study is to assess the attitudes of professional and non-professional healthcare providers with regard to involuntary oral care provision for OIWD who show care-resistant behavior.

## 2. Materials and Methods

### 2.1. Study Design, Sample Size, Setting and Participants

To assess the attitude of professional and non-professional healthcare providers with regard to involuntary oral care provision for OIWD, an online questionnaire was administered between October 2020 and January 2021 to individuals involved in the oral care of one or more OIWD who show care-resistant behavior in the Netherlands. 

Inclusion criteria for participants:

- Professional healthcare providers:
-       Dental professionals:
-                Dentists
-                Dental hygienists
-       Other healthcare professionals:
-              Carers (professionally trained caregivers for ADLs)
-              Nurses
-              Physicians (nursing home physicians (doctors).
- Non-professional healthcare providers:
-       Legal representatives
-       Family caregivers (friends and relatives)

The sample size was calculated by assessing how many participants where needed to be able to determine with 90% reliability that the percentage of participants who would provide involuntary oral care for OIWD per case was representative for the entire population in the Netherlands. An online sample size calculator (AOM Sample Size Calculator (AOM, Zwolle, The Netherlands)) was used. It calculated a sample size of 271 participants. The calculation was based on a margin of error of 5%, a population of 800,000 individuals involved in the care of OIWD in the Netherlands and an outcome (probability that someone gives an specific answer) of 50%. 

The target groups were approached in several ways. First, a message was posted on Facebook (Meta Platforms, Inc., San Francisco, CA, USA) in specific groups for Dutch healthcare providers. If a healthcare provider was interested in participating, they could enroll in the study and complete the questionnaire. Secondly, participants were recruited by requesting, for each profession, specific organizations affiliated with the care of OIWD in the Netherlands (e.g., associations for nurses working with OIWD, associations for dentists working with OIWD, associations for physicians working with OIWD, etc.), to forward the questionnaire to their members, or by contacting members through e-mail addresses on their website.

Thirdly, several nursing homes in the region of the University of Groningen were selected at random and were asked to forward the questionnaire to potential participants (nursing home staff, family members of OIWD living in the nursing home, etc.) by e-mail or by distributing a letter with a QR code to the online questionnaire. Finally, network contacts of the authors were asked to pass the link to the questionnaire to potential participants.

The online questionnaire, which was designed and administered using REDCap^®^ software (Vanderbilt University, TN, USA), consisted of general socio-demographic questions and questions about specific cases involving OIWD with an oral health problem who display care-resistant behavior. Before administering the questionnaire to participants, it was first tested by ten randomly selected healthy individuals working at different departments in the University of Groningen to determine whether or not there were any uncertainties.

Initially, the participant received information about the study, followed by an informed consent form. When the participant completed the questionnaire, they automatically agreed to the terms mentioned in the informed consent form. It took approximately 15 min to complete the questionnaire. 

### 2.2. Demographics and General Questions 

The following characteristics were derived from the general questions:

- Sex (male/female);
- Age (≤35 years/36–50 years/51–65 years/>65 years);
- Involved in the care of OIWD (yes/no);
- Function in care (dentist/dental hygienist/carer/nurse/physician/legal representative/family caregiver/otherwise).

### 2.3. Case Specific Questions

The attitude of participants regarding specific cases concerning oral health problems in OIWD who show care-resistant behavior was derived from answers to questions about specific cases. These cases concerned six different patients with dementia who show care-resistant behavior related to an oral health problem. In all cases, the patient faces a potentially severe health threat if no oral care is provided. A summary of the six cases and the potential health threat is in Table 1. The potential health threats were not shown to participants but are added here to illustrate the potential consequences of not providing oral care in the specific cases. Two questions were asked concerning each case—firstly, whether the participant would assess the oral health problem as potentially harmful; secondly, whether the participant would provide involuntary oral care in that particular situation. 

### 2.4. Study Design and Ethical Considerations

This cross-sectional study was performed at the University of Groningen, whose Medical Ethics Review Committee provided a waiver for this study (file number M20.256419). 

### 2.5. Statistical Analyses

Data were analyzed using SPSS Ver. 26 (IBM Corp., New York, NY, USA). Assessment of harmfulness and willingness to provide involuntary oral care is reported in frequency tables. The differences between assessment of harmfulness and willingness to provide involuntary oral care on ‘function in care’ were evaluated with Pearson Chi-Square tests (Fisher’s exact test was used instead of Pearson Chi-Square test when values were lower than 10 in the frequency table). A *p*-value of ≤ 0.05 was considered statistically significant.

## 3. Results

### 3.1. Participants

**Figure 1:**
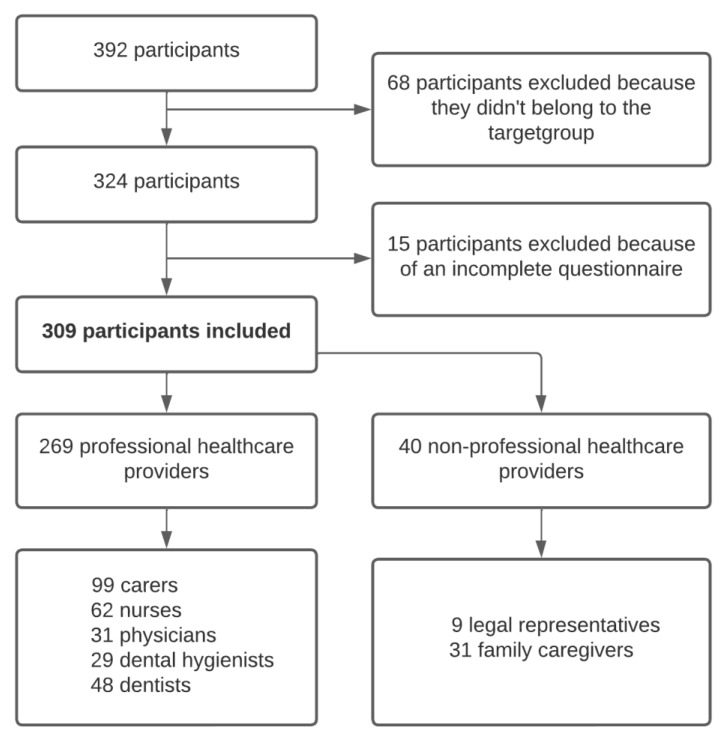
Flowchart of study population.

In total 392 participants responded and filled in the questionnaire, of whom 309 were included in this study: 99 carers, 62 nurses, 31 physicians, 29 dental hygienists, 48 dentists, 9 legal representatives and 31 family caregivers. Their areas of residence were spread widely throughout the Netherlands. The reasons for exclusion were not belonging to the target group (n = 68) and missing data due to an incomplete questionnaire (n = 15) (Figure 1). Most participants were female (84%) and aged between 51 and 65 years (40%).

### 3.2. Assessment of Harmfulness and Willingness to Provide Involuntary Oral Care

An overview of all answers given per specific case is presented in Table 2 and Table 3. The *p*-values of the statistical analyses are presented in Table 4. In all six cases, the proportion of participants who considered oral health problems as potentially harmful (Table 2) is much higher than the proportion of participants who would provide involuntary oral care (Table 3). For example, 93.5% of participants considered Case 6 to be potentially harmful, whereas 63.4% would be willing to provide involuntary oral care in that case. Overall, Cases 3 and 4 were considered to be less potentially harmful than the other four cases (73.1% and 80.9%, respectively). 

### 3.3. Function in Care and Assessment of Harmfulness (Table 2)

Non-professional healthcare providers regarded oral health problems as potentially harmful significantly more often than professional healthcare providers in Cases 1 (97.5% vs. 84.0%, *p* = 0.026) and 3 (90.0% vs. 70.6%, *p* = 0.012). Dental professionals considered an oral health problem as potentially harmful significantly more often than other healthcare professionals in Cases 2 (96.1% vs. 82.8%, *p* = 0.008) and 4 (89.6% vs. 77.1%, *p* = 0.018). Conversely, in Case 3, other healthcare professionals considered an oral health problem as potentially harmful significantly more often than dental professionals (75.5% vs. 58.4%, *p* = 0.005).

### 3.4. Function in Care and Willingness to Provide Involuntary Oral Care (Table 3)

Non-professional healthcare providers would provide involuntary oral care significantly more often than professional healthcare providers in all cases (*p* = 0.003 for Case 1, *p* < 0.001 for Case 2, *p* = 0.001 for Case 3, *p* = 0.009 for Case 4, *p* < 0.001 for Case 5 and *p* = 0.008 for Case 6). Additionally, dental professionals would provide involuntary oral care significantly more often than other healthcare professionals in Cases 1, 2, 4, 5 and 6 (*p* < 0.001 for Case 1, 2, 4 and 6. *p* = 0.001 for Case 5). 

## 4. Discussion

This paper describes the attitudes of Dutch professional and non-professional healthcare providers with regard to involuntary oral care provision for OIWD who show care-resistant behavior. An important finding is the discrepancy between the assessment of oral health problems as potentially harmful and the willingness to provide involuntary oral care in order to prevent further decline of oral health. For example, almost all healthcare providers (93.5%) judged that the oral health problem in Case 6 could harm the patient, whereas around two thirds of the participants (63.4%) were willing to provide involuntary oral care in that case. In other words, participants consider oral care important, but when providing involuntary necessary care, they are often reluctant to do so. It is unknown why this discrepancy exists, but it potentially means that OIWD who show care-resistant behavior do not receive necessary oral care, while evidence indicates that a large proportion (more than half) of the OIWD have (potentially) painful and untreated oral conditions [33]. 

The reluctance to provide involuntary oral care, which is present in all six cases, is consistent with a similar study, where only 21% of nurses in Norwegian nursing homes had considered the use of coercion to help a reluctant patient clean their teeth [9]. In this study, a similar proportion of 25.8% of nurses would be willing to clean a patients’ teeth in an involuntary manner (Case 2). A possible explanation for the reluctance in the present study is that healthcare providers have insufficient knowledge of the actual consequences of poor oral health, and therefore place insufficient important on providing involuntary care. Although they are aware that poor oral health is undesirable, they may not know how harmful it actually can be. A lack of knowledge was seen in all six cases—each case presented an oral health problem that constituted a potential health threat, but in no case did all participants recognize this. 

This possible explanation is consistent with other studies in which a lack of knowledge about oral health and how to provide oral care are reported as barriers to managing oral hygiene of older people in nursing homes [20,21]. Additionally, a possible explanation for the low proportion of Norwegian nurses who would consider involuntary oral care provision could be that most nurses may not consider tooth cleaning important enough to warrant coercive care [9]. 

The potential health threat was least recognized in Cases 3 and 4, where 73.1% and 80.9% of the participants were aware of the potential harm, respectively. In Case 3, only 52.1% of the dentists recognized (potential) harm, although not providing treatment could potentially lead to an acute abscess or infection that needs immediate treatment [29]. This potentially means that dentists would benefit from improved knowledge of oral care provision for OIWD.

A possible explanation for the relatively lower score for Case 4 may be the fact that healthcare providers did not recognize that the patient, grabbing his jaw while eating, is likely to be in pain. Bodily movements can be dementia-related behavior, but they can also be pain-related behavior in individuals with dementia. It may sometimes be difficult to distinguish those two behaviors, but there is strong evidence that especially holding or clutching a body part is a pain indicator for individuals with dementia [30]. Some participants may have been unaware of this. 

A reluctance to provide involuntary care that is based on a lack of knowledge of the consequences of poor oral health may be further strengthened by legal considerations. As stated, Dutch law prohibits involuntary care unless not providing care can cause severe harm [11]. Hence, if healthcare providers do not realize that poor oral health can lead to severe harm, that law may provide further justification for their reluctance. Similarly, Norwegian law states that one of the conditions for providing involuntary care is that not providing care can lead to serious illness, leading to similar outcomes to those in the Netherlands [9]. The question of whether laws regulating involuntary care contribute to a reluctance to provide it requires further research.

Another important result is that non-professional healthcare providers were more willing to provide involuntary oral care when compared to professional healthcare providers. This is consistent with the results of Scheepsman and colleagues, who reported that family or informal caregivers often request or initiate the use of restraints [34]. This can potentially be explained by the attitude of family caregivers providing care for individuals with dementia, as a study of Moermans and colleagues reported that they felt responsible, experienced social pressure for the safety of individuals with dementia, and feared they would be blamed if something adverse happened to the individual with dementia [35]. 

Another pertinent result is that dental professionals would provide involuntary oral care more often than other healthcare professionals, although these other healthcare professionals also assessed oral health problems as (potentially) harmful for the patient. A putative explanation is that dental professionals may only see the patient in the context of oral care, whereas other healthcare professionals, especially carers, see a patient every day and are more emotionally and holistically involved than dental professionals.

Participants’ willingness to provide involuntary oral care varied from case to case. Specifically, the proportion of participants who would provide involuntary oral care in Case 1 was almost twice as high as the proportion of those who would provide involuntary oral care in Case 2 (56.0% vs. 31.1%). This is interesting, as the proportion of participants who assessed the situations in the two cases as (potentially) harmful was almost the same (85.8% and 87.1%). Apparently, the assessment of oral health problems as potentially harmful is not a decisive factor in the willingness to provide involuntary oral care. A possible explanation for the fact that more participants would provide involuntary oral care in Case 1 is that it required less invasive treatment than Case 2. In Case 1, treatment consisted of removing the dentures from the mouth and cleaning them. In Case 2, it consisted of cleaning the patient’s natural teeth, putatively more difficult. 

### Strengths and Limitations

A strength of the present study is its focus—how often and in which circumstances healthcare providers were willing to provide oral care when a patient showed care-resistant behavior. To our knowledge, this is the first study researching involuntary oral care in such a nuanced manner. Another strength is the large number (five) of professional healthcare provider groups in addition to two groups of non-professional healthcare providers. A limitation of the present study is that the only answer options regarding the cases were ‘Yes’ and ‘No’. Because of this, it is unclear how and why healthcare providers made their choices, as many different factors may play a role in the decision-making process. Additionally, the case descriptions did not provide information about the type of involuntary treatment. Participants had to decide whether or not they would be willing to provide involuntary oral care, which could involve different options, such as the patient being restrained, sedated, placed under general anesthetic or the use of side-rails. Therefore, a follow-up study is needed to determine which factors hinder the application of (different forms of) involuntary oral care in OIWD who show care-resistant behavior. Additionally, follow-up studies should be carried out with healthcare providers in other countries who may have different attitudes (and laws) about involuntary oral care in OIWD who show care-resistant behavior. 

## 5. Conclusions

The majority of professional and non-professional healthcare providers in the Netherlands (range: 73.1–93.5%) recognized poor oral health in OIWD who show care-resistant behavior as potentially harmful. However, involuntary oral care is often avoided (range: 36.6–68.9%). 

More research is needed to determine which factors (such as laws, knowledge, time, etc.) hinder the provision of (different forms of) involuntary oral care and to investigate the attitudes of healthcare providers in other countries about involuntary oral care provision in OIWD who show care-resistant behavior.

## Figures and Tables

**Table 1:** Specific cases involving OIWD.

Case 1: A patient shows care-resistant behavior when healthcare providers are trying to remove his dirty dentures in order to clean them. Health threat: Not providing treatment can potentially cause pneumonia due to aspiration of oral debris and oral bacteria, especially in the case of dysphagia [25]. Next, there is a chance of developing problems of the oral mucosa (e.g., candida infection, ulcers, etc.) [26].
Case 2: A patient shows care-resistant behavior when healthcare providers try to clean his natural teeth. Health threat: Not providing oral care can potentially cause gingivitis and increase caries risk which can cause pain [27,28]. Additionally, poor oral health has an impact on general health: it is associated with general health conditions such as cardiovascular disease and diabetic control [17,18,19].
Case 3: A new patient in a nursing home has inflamed gums and there are some broken teeth and root remnants visible while talking. The patient has not seen a dentist for five years. When a dentist is called in for consultation, the dentist states that treatment is needed urgently. However, the patient says he does not want treatment. Health threat: Not providing dental treatment in this case can potentially lead to an acute abscess due to (infected) root remnants, which in turn can cause life threatening complications (e.g., airway obstruction) [29].
Case 4: A patient with natural teeth grabs his jaw every time he eats. However, his eating behavior has not changed. The patient shows care-resistant behavior when someone tries to look into his mouth. Health threat: There is an indication that this patient is in pain while eating as bodily movements are a pain indicator for people with dementia [30]. Pain causes discomfort and behavioral problems. Additionally, there is a general health threat when the pain is caused by an oral health problem [18,29].
Case 5: A patient has dirty teeth, and a large piece of a molar has broken off while eating. The patient shows care-resistant behavior when someone tries to brush his teeth and have a look in his mouth. Health threat: Not providing oral care can (potentially) cause gingivitis and increase caries risk which can cause pain [27,28]. Additionally, poor oral health has an impact on general health: it is associated with general health conditions such as cardiovascular disease and diabetic control [17,18,19].
Case 6: A patient with full dentures grimaces when he chews. The patient has difficulties with eating for a week. The patient shows care-resistant behavior when someone tries to look in his mouth. Health threat: The patient probably has ill-fitting dentures causing mucosal ulcerations. Not providing treatment can (potentially) cause more pain and ongoing ulceration, eventually leading to bone exposure [31]. Not being able to chew properly is a risk for malnutrition [32].

**Table 2:** Participants’ responses to the assessment of harmfulness (Question 1) of Cases 1 to 6.

Position in Care	Case 1		Case 2		Case 3		Case 4		Case 5		Case 6		Total
	Yes n (%)	No n (%)	Yes n (%)	No n (%)	Yes n (%)	No n (%)	Yes n (%)	No n (%)	Yes n (%)	No n (%)	Yes n (%)	No n (%)	n (%)
Professional healthcare providers:	226(84.0%)	43 (16.0%)	233 (86.6%)	36 (13.4%)	190(70.6%)	79 (29.4%)	217 (80.7%)	52 (19.3%)	249 (92.6%)	20 (7.4%)	251 (93.3%)	18 (6.7%)	269 (100.0%)
Dentists	41 (85.4%)	7 (14.6%)	46 (95.8%)	2 (4.2%)	25 (52.1%)	23 (47.9%)	41 (85.4%)	7 (14.6%)	46 (95.8%)	2 (4.2%)	45 (93.8%)	3 (6.3%)	48 (100.0%)
Dental hygienists	25 (86.2%)	4 (13.8%)	28 (96.6%)	1 (3.4%)	20 (69.0%)	9 (31.0%)	28 (96.6%)	1 (3.4%)	29 (100.0%)	0 (0.0%)	29 (100.0%)	0 (0.0%)	29 (100.0%)
Total dental professionals:	66 (85.7%)	11 (14.3%)	74 (96.1%)	3 (3.9%)	45 (58.4%)	32 (41.6%)	69 (89.6%)	8 (10.4%)	75 (97.4%)	2 (2.6%)	74 (96.1%)	3 (3.9%)	77 (100.0%)
Carers	90 (90.9%)	9 (9.1%)	85 (85.9%)	14 (14.1%)	78 (78.8%)	21 (21.2%)	82 (82.8%)	17 (17.2%)	94 (94.9%)	5 (5.1%)	91 (91.9%)	8 (8.1%)	99 (100.0%)
Nurses	50 (80.6%)	12 (19.4%)	52 (83.9%)	10 (16.1%)	48 (77.4%)	14 (22.6%)	46 (74.2%)	16 (25.8%)	55 (88.7%)	7 (11.3%)	57 (91.9%)	5 (8.1%)	62 (100.0%)
Physicians	20 (64.5%)	11 (35.5%)	22 (71.0%)	9 (29.0%)	19 (61.3%)	12 (38.7%)	20 (64.5%)	11 (35.5%)	25 (80.6%)	6 (19.4%)	29 (93.5%)	2 (6.5%)	31 (100.0%)
Total other healthcare professionals:	160 (83.3%)	32 (16.7%)	159 (82.8%)	33 (17.2%)	145 (75.5%)	47 (24.5%)	148 (77.1%)	44 (22.9%)	174 (90.6%)	18 (9.4%)	177 (92.2%)	15 (7.8%)	192 (100.0%)
Non-professional healthcare providers:	39 (97.5%)	1 (2.5%)	36 (90.0%)	4 (10.0%)	36 (90.0%)	4 (10.0%)	33 (82.5%)	7 (17.5%)	40 (100.0%)	0 (0.0%)	38 (95.0%)	2 (5.0%)	40 (100.0%)
Family caregivers	30 (96.8%)	1 (3.2%)	28 (90.3%)	3 (9.7%)	27 (87.1%)	4 (12.9%)	27 (87.1%)	4 (12.9%)	31 (100.0%)	0 (0.0%)	29 (93.5%)	2 (6.5%)	31 (100.0%)
Legal representatives	9 (100.0%)	0 (0.0%)	8 (88.9%)	1 (11.1%)	9 (100.0%)	0 (0.0%)	6 (66.7%)	3 (33.3%)	9 (100.0%)	0 (0.0%)	9 (100.0%)	0 (0.0%)	9 (100.0%)
Total:	265 (85.8%)	44 (14.2%)	269 (87.1%)	40 (12.9%)	226 (73.1%)	83 (26.9%)	250 (80.9%)	59 (19.1%)	289 (93.5%)	20 (6.5%)	289 (93.5%)	20 (6.5%)	309 (100.0%)

**Table 3:** Participants’ responses the willingness to provide involuntary oral care (Question 2) of Cases 1 to 6.

Position in Care	Case 1		Case 2		Case 3		Case 4		Case 5		Case 6		Total
	Yes n (%)	No n (%)	Yes n (%)	No n (%)	Yes n (%)	No n (%)	Yes n (%)	No n (%)	Yes n (%)	No n (%)	Yes n (%)	No n (%)	n (%)
Professional healthcare providers:	142(52.8%)	127 (47.2%)	74(27.5%)	195 (72.5%)	75(27.9%)	194 (72.1%)	136(50.6%)	133 (49.4%)	119(44.2%)	150 (55.8%)	163(60.6%)	106 (39.4%)	269 (100.0%)
Dentists	33 (68.8%)	15 (31.3%)	21 (43.8%)	27 (56.3%)	11 (22.9%)	37 (77.1%)	36 (75.0%)	12 (25.0%)	28 (58.3%)	20 (41.7%)	40 (83.3%)	8 (16.7%)	48 (100.0%)
Dental hygienists	21 (72.4%)	8 (27.6%)	15 (51.7%)	14 (48.3%)	11 (37.9%)	18 (62.1%)	22 (75.9%)	7 (24.1%)	18 (62.1%)	11 (37.9%)	25 (86.2%)	4 (13.8%)	29 (100.0%)
Total dental professionals:	54 (70.1%)	23 (29.9%)	36 (46.8%)	41 (53.2%)	22 (28.6%)	55 (71.4%)	58 (75.3%)	19 (24.7%)	46 (59.7%)	31 (40.3%)	65 (84.4%)	12 (15.6%)	77 (100.0%)
Carers	47 (47.5%)	52 (52.5%)	19 (19.2%)	80 (80.8%)	20 (20.2%)	79 (79.8%)	36 (36.4%)	63 (63.6%)	35 (35.4%)	64 (64.6%)	44 (44.4%)	55 (55.6%)	99 (100.0%)
Nurses	28 (45.2%)	34 (54.8%)	16 (25.8%)	46 (74.2%)	26 (41.9%)	36 (58.1%)	29 (46.8%)	33 (53.2%)	28 (45.2%)	34 (54.8%)	35 (56.5%)	27 (43.5%)	62 (100.0%)
Physician	13 (41.9%)	18 (58.1%)	3 (9.7%)	28 (90.3%)	7 (22.6%)	24 (77.4%)	13 (41.9%)	18 (58.1%)	10 (32.3%)	21 (67.7%)	19 (61.3%)	12 (38.7%)	31 (100.0%)
Total other healthcare professionals:	88 (45.8%)	104 (54.2%)	38 (19.8%)	154 (80.2%)	53 (27.6%)	139 (72.4%)	78 (40.6%)	114 (59.4%)	73 (38.0%)	119 (62.0%)	98 (51.0%)	94 (49.0%)	192 (100.0%)
Non-professional healthcare providers:	31 (77.5%)	9 (22.5%)	22 (55.0%)	18 (45.0%)	22 (55.0%)	18 (45.0%)	29 (72.5%)	11 (27.5%)	30 (75.0%)	10 (25.0%)	33 (82.5%)	7 (17.5%)	40 (100.0%)
Family caregivers	23 (74.2%)	8 (25.8%)	14 (45.2%)	17 (54.8%)	14 (45.2%)	17 (54.8%)	21 (67.7%)	10 (32.3%)	21 (67.7%)	10 (32.3%)	24 (77.4%)	7 (22.6%)	31 (100.0%)
Legal representatives	8 (88.9%)	1 (11.1%)	8 (88.9%)	1 (11.1%)	8 (88.9%)	1 (11.1%)	8 (88.9%)	1 (11.1%)	9 (100.0%)	0 (0.0%)	9 (100.0%)	0 (0.0%)	9 (100.0%)
Total:	173 (56.0%)	136 (44.0%)	96 (31.1%)	213 (68.9%)	97 (31.4%)	212 (68.6%)	165 (53.4%)	144 (46.6%)	149 (48.2%)	160 (51.8%)	196 (63.4%)	113 (36.6%)	309 (100.0%)

**Table 4:** *p*-values of statistical analyses of Cases 1 to 6.

Position in Care	Case 1		Case 2		Case 3		Case 4		Case 5		Case 6	
	AH *	IC **	AH *	IC **	AH *	IC **	AH *	IC **	AH *	IC **	AH *	IC **
Professional healthcare providers vs. non professionals healthcare providers	**0.026** ^f^	**0.003** ^c^	0.800 ^f^	**<0.001** ^c^	**0.012** ^f^	**0.001** ^c^	1.000 ^f^	**0.009** ^c^	0.088 ^f^	**<0.001** ^c^	1.000 ^f^	**0.008** ^f^
Dental professionals vs. other healthcare professionals	0.630 ^c^	**<0.001** ^c^	**0.008** ^f^	**<0.001** ^c^	**0.005** ^c^	0.873 ^c^	**0.018** ^f^	**<0.001** ^c^	0.071 ^f^	**0.001** ^c^	0.293 ^f^	**<0.001** ^c^
Dentists vs. dental hygienists	1.000 ^f^	0.801 ^f^	1.000 ^f^	0.497 ^c^	0.161 ^f^	0.158 ^c^	0.246 ^f^	1.000 ^f^	0.524 ^f^	0.746 ^c^	0.286 ^f^	1.000 ^f^
Carers vs. nurses vs. physicians	**0.002** ^f^	0.851 ^c^	0.165 ^f^	0.186 ^c^	0.136 ^c^	**0.011** ^f^	0.093 ^c^	0.444 ^c^	**0.044** ^f^	0.353 ^c^	1.000 ^f^	0.161 ^c^

* AH: assessment of harmfulness; ** IC: willingness to provide involuntary oral care; ^f^: Fisher’s exact test; ^c^: Pearson Chi-Square test; statistically significant *p*-values in bold.

## Data Availability

Data of the statistical analyses are available on request.
